# An Unusual Case of Ototoxicity with Use of Oral Vancomycin

**DOI:** 10.1155/2018/2980913

**Published:** 2018-07-03

**Authors:** Umut Gomceli, Srija Vangala, Cosmina Zeana, Paul J. Kelly, Manisha Singh

**Affiliations:** Department of Internal Medicine, Bronx Lebanon Hospital Center, Bronx, NY, USA

## Abstract

**Introduction:**

Systemic absorption of oral vancomycin is poor due to the size of the molecule and its pharmacokinetics. It has an elimination half life of 5–11 hours in patients with normal renal function. We present a rare case of ototoxicity after oral vancomycin administration and detectable serum vancomycin levels 24 hours after cessation of vancomycin.

**Case Presentation:**

A 42-year-old woman with a history of hypertension, diabetes mellitus, and previously treated *Clostridium difficile* colitis presented with abdominal pain and diarrhea for two weeks. *Clostridium difficile* infection was confirmed by PCR, and at the time of diagnosis and initiation of therapy, the patient had normal renal function. Vancomycin was initiated at a dose of 125 mg po q6h. After the third dose of oral vancomycin, the patient reported new symptoms of lightheadedness, sensations of “buzzing” and whistling of bilateral ears, and decreased perception of hearing described as “clogged ears.” The patient reported to the emergency department the next day due to worsening of these symptoms, and vancomycin dosing was reduced to every 8 hours; however, the patient reported the auditory symptoms persisted. On day three, vancomycin was discontinued with gradual resolution of symptoms over the next 12 hours. On day four, a serum random vancomycin level obtained 24 hours after the last dose was detectable at 2 mcg/dl. Temporal association of the patient's symptoms and improvement with cessation of therapy along with a detectable vancomycin level indicates systemic absorption of oral vancomycin with subsequent ototoxicity.

**Discussion:**

The potential for absorption of oral vancomycin is not well described and is attributed to compromised intestinal epithelium allowing for increased drug absorption. Few studies suggested that oral vancomycin may result in therapeutic or even potentially toxic levels of serum vancomycin in patients with impaired renal function. Ototoxicity may be a transient or permanent side effect of vancomycin therapy and is related to high serum levels. Symptoms usually resolve after decreasing the dose or cessation of vancomycin. No detectable serum vancomycin levels were found in 98% of the patients treated with oral vancomycin in a prospective study. The described case is unusual because despite normal renal function, the patient still developed ototoxicity, and systemic absorption of the drug was confirmed with a measurable vancomycin level approximately 24 hours after the drug was stopped. Additionally, the only other medication prescribed to the patient at the time of vancomycin administration was metformin at a dose of 500 mg po bid which has no known idiosyncratic interactions potentiating adverse side effects to vancomycin. This case reflects that some patients may be more susceptible to increased systemic absorption via the oral route, and the possibility for ototoxicity should be considered and discussed with patients while prescribing oral vancomycin.

## 1. Introduction

*Clostridium difficile* infection (CDI) has become an increasingly common infection with the widespread use of the antibiotic therapy [1]. It is the most common cause of nosocomial diarrhea, yet the incidence of CDI in the community is also on the rise [2, 3]. Metronidazole is recommended for the treatment of patients with mild to moderate CDI and oral vancomycin for the treatment of patients with severe CDI and recurrent disease [2]. Oral vancomycin is predicted to be poorly absorbed from the gastrointestinal tract based on pharmacokinetic data [4]. However, prior reports have documented that oral vancomycin therapy may produce detectable serum concentrations in patients with severe colitis and renal failure [5, 6]. There are no reports of ototoxicity as a complication following administration of oral vancomycin. We report the first case of ototoxicity after administration of oral vancomycin in a patient with normal renal function.

## 2. Case Presentation

A 42-year-old woman with history of hypertension and diabetes mellitus presented to the outpatient clinic with abdominal pain and diarrhea for two weeks. The patient was recently treated with clindamycin for sinusitis. On examination, the patient appeared comfortable, afebrile, and had normal vital signs. There was mild tenderness reported on abdominal palpation, and the remainder of the physical examination was unremarkable. *Clostridium difficile* infection was confirmed by a positive stool toxin B PCR, and the patient was started on treatment with metronidazole. Due to complaints of nausea on day three of metronidazole use, the treatment was changed to oral vancomycin at 125 mg every 6 hours. At the time of initiation of therapy, the patient had creatinine of 0.6 mg/dL. After the third day of oral vancomycin, the patient reported new symptoms of lightheadedness, sensations of “buzzing and whistling” in both ears, as well as decreased perception of hearing described as “clogged ears.” The patient presented to the emergency department (ED) due to worsening of these symptoms, and the vancomycin dose was reduced to 125 mg every 8 hours. However, the reported symptoms persisted, and on day 5 of therapy, vancomycin was discontinued in the outpatient clinic. A random vancomycin level obtained 24 hours after the last dose of vancomycin, resulted as 2 mcg/mL. The patient's symptoms were also reported to be resolved within 24 hours after discontinuation of therapy. The temporal association of the patient's symptoms and improvement with cessation of therapy along with a detectable vancomycin level indicates systemic absorption of oral vancomycin with associated ototoxicity.

## 3. Discussion

CDI is a well-established cause of nosocomial diarrhea in hospitals and long-term care facilities leading to a large burden of cost [1]. The epidemiology of *C. difficile*-associated disease has changed in the last decade, and it is now an important pathogen in the community [1, 3]. In addition to an increasing incidence and virulence of infection, almost one fourth of patients have recurrent disease [7].

Vancomycin appears to be more efficacious than metronidazole for treatment of CDI [8]. Current treatment guidelines recommend use of oral vancomycin for severe disease and after the first recurrence of infection [2]. Poor absorption of oral vancomycin as predicted by pharmacokinetics forms the basis for use of oral vancomycin for colitis due to *Clostridium difficile* [5]. However, there are conflicting clinical data regarding the systemic absorption after oral administration. There are reported cases of detectable serum levels of vancomycin after oral administration in the setting of severe colitis and renal insufficiency [5, 9]. In one series of 10 cases treated with oral vancomycin, 4 had detectable levels ranging 1.0–3.1 mg/L. Out of the 4 patients, 1 had renal insufficiency [10]. In a prospective observational study including 85 patients, detectable vancomycin levels were observed in 68% cases. The observed risk factors for systemic exposure of the drug included ICU admission, over 10 days of therapy, presence of severe CDI, renal dysfunction, inflammatory conditions of the GI tract, and concomitant use of vancomycin retention enemas [6]. There are also reports indicating lack of systemic absorption of oral vancomycin [11]. In a recent pilot study including 8 children, 7 with inflammatory bowel disease and one with acute kidney injury, none had detectable levels of vancomycin after enteral administration [12]. A prospective study including 57 adults showed no detectable serum vancomycin in 98% of the patients treated with oral vancomycin. Furthermore, despite the fact that renal excretion is the dominant route of vancomycin clearance, systemic absorption did not occur even in patients with renal insufficiency [13]. Transient or permanent ototoxicity has rarely been described as a side effect of vancomycin therapy and is related to high serum vancomycin levels [4]. Symptoms usually resolve after decrease in dose or cessation of vancomycin. There is no reported case of ototoxicity associated with use of oral vancomycin. Our case is unusual because despite the absence of renal failure and other known risk factors for systemic exposure to vancomycin, the patient developed symptoms suggestive of ototoxicity. The patient was not concurrently taking other medications known to causing ototoxicity or with potential pharmacokinetic interactions with vancomycin. Systemic absorption was confirmed by a measurable vancomycin level of 2.0 mg/mL approximately 24 hours after the drug was stopped. Due to the time lag in obtaining the drug level, the peak serum level of vancomycin could have been significantly higher than the measured level. Using the adverse drug reaction probability calculation method described by Naranjo et al., our case would be considered a “probable” drug reaction due to oral vancomcyin [14].

## 4. Conclusion

Our case reflects that ototoxicity due to vancomycin can develop after oral administration in the absence of renal impairment or other reported risk factors for systemic absorption. The possibility of systemic absorption and possible ototoxicity should be considered and discussed with patients when prescribing oral vancomycin.

## References

[1] Evans C. T., Safdar N. (2015). Current trends in the epidemiology and outcomes of *Clostridium difficile* infection. *Clinical Infectious Diseases*.

[2] Cohen S. H., Gerding D. N., Johnson S. (2010). Clinical practice guidelines for *Clostridium difficile* infection in adults: 2010 update by the Society for Healthcare Epidemiology of America (SHEA) and the Infectious Diseases Society of America (IDSA). *Infection Control & Hospital Epidemiology*.

[3] Chitnis A. S., Holzbauer S. M., Belflower R. M. (2013). Epidemiology of community-associated *Clostridium difficile* infection, 2009 through 2011. *JAMA Internal Medicine*.

[4] Moellering R. C. (1984). Pharmacokinetics of vancomycin. *Journal of Antimicrobial Chemotherapy*.

[5] Spitzer P. G., Eliopoulos G. M. (1984). Systemic absorption of enteral vancomycin in a patient with pseudomembranous colitis. *Annals of Internal Medicine*.

[6] Pettit N. N., DePestel D. D., Fohl A. L., Eyler R., Carver P. L. (2015). Risk factors for systemic vancomycin exposure following administration of oral vancomycin for the treatment of *Clostridium difficile* infection. *Pharmacotherapy*.

[7] Kelly C. P., Lamont J. T. (2008). *Clostridium difficile*-more difficult than ever. *New England Journal of Medicine*.

[8] Zar F. A., Bakkanagari S. R., Moorthi K. M. (2007). A comparison of vancomycin and metronidazole for the treatment of *Clostridium difficile*-associated diarrhea, stratified by disease severity. *Clinical Infectious Diseases*.

[9] Matzke G. R., Halstenson C. E., Olson P. L. (1987). Systemic absorption of oral vancomycin in patients with renal insufficiency and antibiotic-associated colitis. *American Journal of Kidney Diseases*.

[10] Armstrong C. J., Wilson T. S. (1995). Systemic absorption of vancomycin. *Journal of Clinical Pathology*.

[11] Tedesco F., Markham R., Gurwith M., Christie D., Bartlett J. G. (1978). Oral vancomycin for antibiotic-associated pseudomembranous colitis. *The Lancet*.

[12] Antoon J. W., Hall M., Metropulos D., Steiner M. J., Jhaveri R., Lohr J. A. (2016). A prospective pilot study on systemic absorption of oral vancomycin in children with colitis. *Journal of Pediatric Pharmacology and Therapeutics*.

[13] Rao S., Kupfer Y., Pagala M., Chapnick E., Tessler S. (2011). Systemic absorption of oral vancomycin in patients with *Clostridium difficile* infection. *Scandinavian Journal of Infectious Diseases*.

[14] Naranjo C. A., Busto U., Sellers E. M. (1981). A method for estimating the probability of adverse drug reactions. *Clinical Pharmacology and Therapeutics*.

